# Exercise-Induced Fatigue Impairs Bidirectional Corticostriatal Synaptic Plasticity

**DOI:** 10.3389/fncel.2018.00014

**Published:** 2018-01-25

**Authors:** Jing Ma, Huimin Chen, Xiaoli Liu, Lingtao Zhang, Decai Qiao

**Affiliations:** College of Physical Education and Sports, Beijing Normal University, Beijing, China

**Keywords:** exercise-induced fatigue, corticostriatal pathway, long-term potentiation, long-term depression, glutamate release

## Abstract

Exercise-induced fatigue (EF) is a ubiquitous phenomenon in sports competition and training. It can impair athletes’ motor skill execution and cognition. Corticostriatal synaptic plasticity is considered to be the cellular mechanism of movement control and motor learning. However, the effect of EF on corticostriatal synaptic plasticity remains elusive. In the present study, using field excitatory postsynaptic potential recording, we found that the corticostriatal long-term potentiation (LTP) and long-term depression (LTD) were both impaired in EF mice. To further investigate the cellular mechanisms underlying the impaired synaptic plasticity in corticostriatal pathway, whole-cell patch clamp recordings were carried out on striatal medium spiny neurons (MSNs). MSNs in EF mice exhibited increased spontaneous excitatory postsynaptic current (sEPSC) frequency and decreased paired-pulse ratio (PPR), while with normal basic electrophysiological properties and normal sEPSC amplitude. Furthermore, the N-methyl-D-aspartate (NMDA)/α-amino-3-hydroxy-5-methyl-4-isoxazolepropionate (AMPA) ratio of MSNs was reduced in EF mice. These results suggest that the enhanced presynaptic glutamate (Glu) release and downregulated postsynaptic NMDA receptor function lead to the impaired corticostriatal plasticity in EF mice. Taken together, our findings for the first time show that the bidirectional corticostriatal synaptic plasticity is impaired after EF, and suggest that the aberrant corticostriatal synaptic plasticity may be involved in the production and/or maintenance of EF.

## Introduction

Exercise-induced fatigue (EF) is a reduction in maximal voluntary muscle force that results from intense and prolonged exercise (Gandevia, 2001). EF is a complex phenomenon influenced by both peripheral and central factors. It is not entirely due to the peripheral changes at the level of muscle, an inability of the central nervous system (CNS) to drive skeletal muscle effectively is also involved, which is known as “central fatigue” (Meeusen and Piacentini, 2003; Nybo and Secher, 2004; Meeusen et al., 2006). However, the neuronal and molecular mechanisms of central fatigue have not been well elucidated.

Striatum, the primary input nucleus of basal ganglia, plays a crucial role in action control and motor skill learning (Di Filippo et al., 2009; Zhai et al., 2017). In the striatum, the vast majority of neurons are GABAergic medium spiny neurons (MSNs), which account for over 95% of striatal neurons, while the cholinergic and GABAergic interneurons account for less than 5% (Kreitzer, 2009). MSNs receive convergent glutamatergic inputs from the cerebral cortex and thalamus, as well as dopaminergic inputs from the substantia nigra pars compacta (Bolam et al., 2000; Kreitzer and Malenka, 2008). Repetitive activation of cortical inputs can induce long-term changes of synaptic plasticity in the corticostriatal pathway. Long-term potentiation (LTP) and long-term depression (LTD), the two classic forms of synaptic plasticity, have both been described in the corticostriatal pathway, and LTD is the predominant form (Partridge et al., 2000; Ronesi and Lovinger, 2005). Corticostriatal synaptic plasticity plays an important role in regulating the excitatory inputs to the basal ganglia, and is believed to be the cellular substrate for voluntary motor control, motor learning and habit formation (Reynolds and Wickens, 2002; Yin and Knowlton, 2006). Movement disorders including Parkinson’s disease (PD; Bagetta et al., 2010; Paillé et al., 2010), Huntington’s disease (HD; Dalbem et al., 2005; Picconi et al., 2006) and dystonia (Martella et al., 2009) have shown to be associated with abnormal corticostriatal synaptic plasticity.

Extensive studies on humans suggest that appropriate exercise is beneficial to maintain brain health, synaptic plasticity and cognitive function (Cotman and Berchtold, 2002; Intlekofer and Cotman, 2013; Fernandes et al., 2017). Animal experiments also demonstrate that voluntary exercise can increase neuronal survival and reduce brain damage, enhance learning, LTP and cognitive function (Stummer et al., 1994; Escorihuela et al., 1995; van Praag et al., 1999; Carro et al., 2001). However, intense and prolonged exercise without sufficient recovery may lead to fatigue, which not only affects the motor skill execution (Aune et al., 2008), but also impairs the synaptic plasticity and cognitive function, such as attention, information processing and decision making (Thomson et al., 2009; Moore et al., 2012). For example, Connell et al. (2016a,b) found that EF reduced the velocity of saccadic eye movements, impaired the oculomotor control and attention. Thomson et al. (2009) found that athletes’ decision-making time decreased and decision-making errors increased after EF. Sun et al. (2017) showed that high-intensity treadmill running impaired the mice’ spatial memory and hippocampal synaptic plasticity. Corticostriatal synaptic plasticity plays a crucial role in modulating motor activity, and is the cellular basis of motor skill learning and cognitive performance (Calabresi et al., 1996; Mahon et al., 2004; Chepkova et al., 2013). However, to our knowledge, no studies have reported the effect of EF on corticostriatal synaptic plasticity.

In the present study, by using *in vitro* electrophysiological techniques, we examined whether corticostriatal synaptic plasticity was affected in response to EF. Our results showed that N-methyl-D-aspartate (NMDA) receptor-dependent LTP and endocannabinoid-dependent LTD (eCB-LTD) in corticostriatal pathway were both impaired in EF mice. Further, we found that the basic electrophysiological properties and spontaneous excitatory postsynaptic current (sEPSC) amplitude of MSNs were normal, but sEPSC frequency was increased, paired-pulse ratio (PPR) was decreased and NMDA/α-amino-3-hydroxy-5-methyl-4-isoxazolepropionate (AMPA) ratio was decreased in the MSNs of EF mice. These results suggest that in the striatum the probability of presynaptic glutamate (Glu) release is enhanced and postsynaptic NMDA receptor function is impaired after EF.

## Materials and Methods

### Animals

Male C57BL/6 mice (8 weeks old, Beijing Vital River Laboratory Animal Technology Company, Beijing, China) were housed under artificial light (12 h light/dark cycle, lights on at 7:00 AM) at 22 ± 2°C. Food and water were provided *ad libitum*. This study was carried out in accordance with the recommendations of Animal Care and Use Committee of Beijing Normal University. The protocol was approved by the Animal Care and Use Committee of Beijing Normal University.

### Fatiguing Exercise Protocol

The C57BL/6 male mice were randomly divided into two groups: EF group (EF) and control group (Control). The fatiguing exercise protocol was adapted from Rosa et al. (2008) and Costa et al. (2014). In brief, exercised mice were initially acclimated to the treadmill (DSPT-202, HangZhou DuanShi, China) for 15-min daily with 10 m/min speed for three successive days (adaptation stage). The individual maximum velocity was tested as follows: 3-min of warm-up at 5 m/min, 1 min with treadmill at 10 m/min followed by further progressive increases of 1 m/min every minute until mice reached exhaustion, and 3-min of cool-down at 5 m/min. Subsequently, the exercised mice were submitted to the seven successive days of intense and exhaustive exercise: 3-min of warm-up at 5 m/min, running at 85% of maximum velocity until mice reached exhaustion, 3-min of cool-down at 5 m/min. The state of animals’ fatigue was identified by a 15-s refusal of mice to run even after gentle hand prodding. Electrical shock was avoided to drive mice running, because this would have added undue stress, which is not associated with exercise. Control mice were exposed to the same environmental conditions (handling, treadmill motor noise) as exercised mice.

### Preparation of Corticostriatal Slices

After fatiguing exercise, mice were anesthetized with chloral hydrate (400 mg/kg, intraperitoneal injection) and decapitated. The brains were immediately removed and chilled in ice-cold modified artificial cerebrospinal fluid (mACSF) containing (in mM): 213 Sucrose, 2.5 KCl, 1.25 NaH_2_PO_4_, 26 NaHCO_3_, 10 D-glucose, 2 MgSO_4_ and 2 CaCl_2_. Coronal corticostriatal slices (400 μm thick for field excitatory postsynaptic potentials (fEPSPs) recording and 300 μm thick for whole-cell recording) were cut with a vibratome (VT1000S, Leica, Germany) in ice-cold mACSF. Fresh slices were then transferred to a chamber containing regular ACSF (in mM, 126 NaCl, 2.5 KCl, 1.25 NaH_2_PO_4_, 26 NaHCO_3_, 25 D-glucose, 2 MgSO_4_ and 2 CaCl_2_) at 30 °C for at least 1 h before recording. All solutions were saturated with 95% O_2_/5% CO_2_.

### Field Excitatory Postsynaptic Potentials Recording

Corticostriatal slices (400 μm thick) were transferred to a recording chamber and submerged in continuously flowing artificial cerebrospinal fluid (ACSF; 2–3 ml/min) bubbled with 95% O_2_/5% CO_2_ at room temperature. During fEPSPs recording, a tungsten-stimulating electrode was placed in the white matter, which gives rise to broad and diffuse activation of striatal neurons and picrotoxin (50 μM) was added to ACSF to suppress the synaptic current mediated by GABA_A_ receptors. Extracellular fEPSPs in dorsolateral striatum were recorded with ACSF-filled glass microelectrode (4–8 MΩ) in current-clamp by MultiClamp 700B amplifier (Molecular Devices, Sunnyvale, CA, USA). Test responses were elicited at 0.033 Hz. For LTP recording, Mg^2+^ were omitted from ACSF to better disclose the NMDA receptor channels. After recording a stable baseline for at least 15 min, LTP was induced by high-frequency stimulation (HFS, 100 Hz for 3 s, three trains, 20 s inter-train interval; Paillé et al., 2010). LTD was induced by the same HFS protocol (100 Hz for 3 s, three trains, 20 s inter-train interval), but in the presence of Mg^2+^ (Paillé et al., 2010). The magnitude of LTP and LTD were calculated by comparing the average fEPSPs amplitude between 35 min and 45 min to that of the 15 min baseline.

### Whole-cell Patch Clamp Recording

The candidate MSNs in dorsolateral striatum were identified by using infrared-differential interference contrast video microscopy (BX50WI, Olympus, Japan). Patch pipettes (3–5 MΩ) were made from borosilicate glass capillaries pulled on a P-97 micropipette puller (Sutter Instruments, Novato, CA, USA). Whole-cell patch clamp recordings were performed in gap-free acquisition mode with a sampling rate of 10 kHz and low-pass filtered at 2 kHz, using MultiClamp 700B amplifier, Digidata 1550 and pClamp 10.5 software (Molecular Devices, Sunnyvale, CA, USA). Access resistance was continuously monitored during the experiments. Cells were excluded if the access resistance was >25 MΩ.

Action potentials were elicited by injection of a series of hyperpolarizing and depolarizing current steps (500 ms, from −100 pA to +500 pA in 20 pA steps). The resting membrane potential was recorded under the “gap-free” model, and the average of at least 5-min steady membrane potential was used for statistical analysis. Input resistance was calculated as the slope of a V-I plot, measuring the change of peak voltage resulting from a series of 500 ms current injections from −100 pA to +40 pA. MSNs were identified by their medium-sized somas and characteristic electrophysiological properties, including hyperpolarized resting membrane potential, inward rectification in the hyperpolarizing direction, delayed action potential discharge with respect to onset of current injection, and low input resistance (Pawlak and Kerr, 2008). For sEPSC recording, MSNs were voltage-clamped at −70 mV in the presence of picrotoxin (50 μM). Internal electrode fluid for resting membrane potential, action potential and sEPSC recording contained (in mM) 140 K-gluconate, 3 KCl, 2 MgCl_2_, 0.2 EGTA, 10 HEPES, 2 ATP (Na^+^ salt), with pH adjusted to 7.2 by KOH, and osmotic pressure adjusted to 280–290 mOsmol/L.

For evoked excitatory postsynaptic current (eEPSC) recording, picrotoxin (50 μM) was added in the ACSF, a tungsten-stimulating electrode was placed in the white matter, and MSNs in the dorsolateral striatum were chosen to record. Stimulus intensity was adjusted to elicit EPSC amplitude between 200 pA and 400 pA. PPR was elicited by paired stimulus with various inter-stimulus intervals (ISI; 20, 50, 100, 200 and 500 ms), and calculated as the ratio of the second EPSC amplitude to the first EPSC (EPSC_2_/EPSC_1_). NMDA/AMPA ratio was calculated as the ratio of the EPSC recorded at + 40 mV, 50 ms after afferent stimulation (NMDA receptor-mediated EPSC) to the peak EPSC recorded at −70 mV (AMPA receptor-mediated EPSC). The internal electrode fluid contained (in mM) 120 CsMeSO_3_, 15 CsCl, 8 NaCl, 10 TEA, 10 HEPES, 5 QX-314, 0.2 EGTA, 2 ATP (Mg^2+^ salt), 0.3 GTP (Na^+^ salt), with PH adjusted to 7.3 by CsOH, and osmotic pressure adjusted to 290–300 mOsmol/L.

### Statistical Analysis

All numerical data were expressed as mean ± SEM. Student’s *t*-test was used to determine the statistical significance between means. sEPSC data were analyzed by using Mini Analysis software (Synaptosoft, Fort Lee, NJ, USA), with an amplitude threshold of 5 pA. Cumulative distributions were generated by using 100 consecutive sEPSCs from each cell, and averaged across all cells (Peng et al., 2009; Ma et al., 2015). Kolmogorov-Smirnov test was used to compare cumulative distributions of sEPSC. The significance level was set at *p* < 0.05.

## Results

### NMDA Receptor-Dependent LTP Is Impaired in Exercise-Induced Fatigue Mice

**Figure 1:**
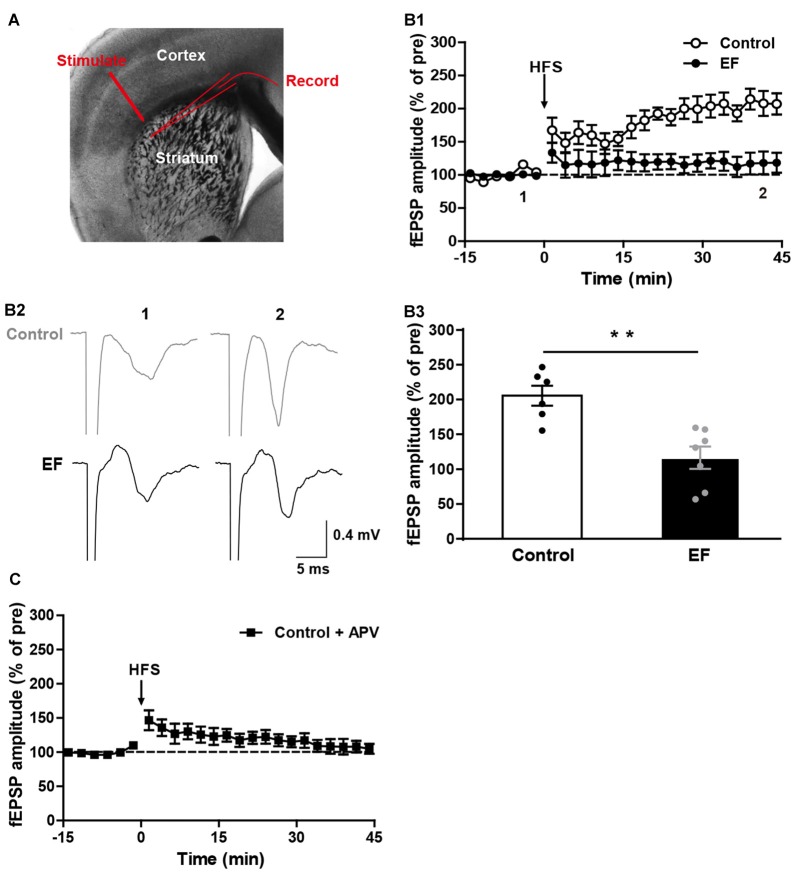
N-methyl-D-aspartate (NMDA) receptor-dependent long-term potentiation (LTP) was impaired in exercise-induced fatigue (EF) mice. **(A)** Image of a live coronal corticostriatal slice taken with a 4× objective showing the placement of stimulating and recording electrodes.** (B1)** LTP induced by high-frequency stimulation (HFS) in Mg^2+^-free artificial cerebrospinal fluid (ACSF) was impaired in EF mice. **(B2)** Sample traces (average of 20 consecutive sweeps) taken at baseline (1) and the last 10 min recording (2). **(B3)** The average field excitatory postsynaptic potential (fEPSP) amplitudes of the last 10 min recording (35–45 min in **A1**) were used for the plot.** (C)** Corticostriatal LTP in control mice was blocked by NMDA receptor antagonist APV (50 μM). All data are showed as mean ± SEM. Student’s *t*-test, **p < 0.01.

Fatigue is known to have difficulty in initiating or sustaining voluntary movements, and is accompanied by deterioration in exercise performance (Tanaka et al., 2008). Corticostriatal synaptic plasticity is involved in voluntary motor control and motor learning (Reynolds and Wickens, 2002; Yin and Knowlton, 2006). To investigate whether EF affects the corticostriatal plasticity, we examined the LTP and LTD in EF mice and control mice. As shown in Figure 1A, stimulating electrode was placed in the white matter, and recording electrode was placed in the dorsolateral striatum. HFS (100 Hz for 3 s, three trains, 20 s inter-train interval) of cortical afferents in Mg^2+^-free ACSF induced robust corticostriatal LTP in control mice, but not in EF mice (Figure 1B, Control: 205.60 ± 14.34% of pre at 35–45 min, *n* = 6 slices/4 mice; EF: 116.50 ± 15.77% of pre at 35–45 min, *n* = 7 slices/5 mice; Student’s *t*-test, *p* < 0.01). In addition, the corticostriatal LTP in control mice was blocked by NMDA receptor antagonist APV (50 μM; Figure 1C, Control + APV: 107.29 ± 9.11% of pre at 35–45 min, *n* = 9 slices/5 mice), suggesting that the corticostriatal LTP induced under our condition is NMDA receptor-dependent. These results indicate that EF impairs NMDA receptor-dependent LTP in the corticostriatal pathway.

### Endocannabinoid-Dependent LTD Is Impaired in Exercise-Induced Fatigue Mice

**Figure 2:**
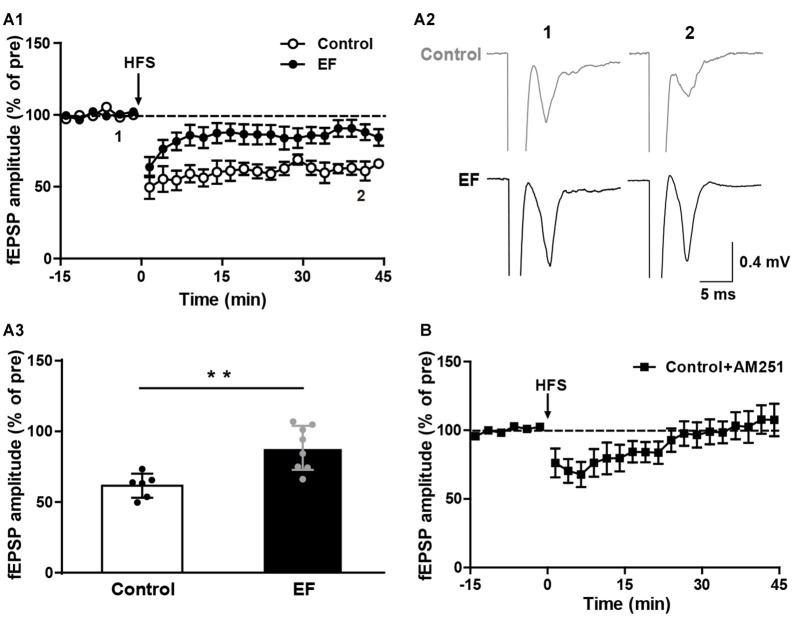
Endocannabinoid (eCB)-dependent long-term depression (LTD) was impaired in EF mice. **(A1)** LTD induced by HFS in Mg^2+^-containing ACSF was impaired in EF mice. **(A2)** Sample traces (average of 20 consecutive sweeps) taken at baseline (1) and the last 10 min recording (2). **(A3)** The average fEPSP amplitudes of the last 10 min recording (35–45 min in **A1**) were used for the plot. **(B)** Corticostriatal LTD in control mice was blocked by CB1 receptor antagonist AM251 (1 μM). All data are showed as mean ± SEM. Student’s *t*-test, **p < 0.01.

The striatal LTD is modulated by endocannabinoid (eCB; Kreitzer and Malenka, 2008; Kano et al., 2009). Endocannabinoid-dependent LTD (eCB-LTD) is the predominant form of synaptic plasticity in the corticostraital pathway. eCBs are released by the postsynaptic neurons and act as retrograde messengers to activate presynaptic cannabinoid type-1 (CB1) receptors, leading to reduced neurotransmitter release probability and thus depressing neurotransmission (Kano et al., 2009; Wu et al., 2015). We found that in normal Mg^2+^-containing ACSF, HFS of corticostriatal fibers induced remarkable LTD in control mice, but in EF mice the LTD was significantly impaired (Figure 2A, Control: 61.56 ± 3.48% of pre at 35–45 min, *n* = 6 slices/4 mice; EF: 88.36 ± 5.52% of pre at 35–45 min, *n* = 8 slices/5 mice; Student’s *t*-test, *p* < 0.01). Applying the CB1 receptor antagonist AM251 (1 μM) in ASCF blocked LTD in control mice (Figure 2B, Control + AM251: 105.25 ± 10.74% of pre at 35–45 min, *n* = 7 slices/4 mice), which confirms that this LTD is eCB-dependent. Taken together, these results indicate that EF impairs eCB-LTD in the corticostriatal pathway.

### Basic Electrophysiological Properties of MSNs Are Normal in Exercise-Induced Fatigue Mice

**Figure 3:**
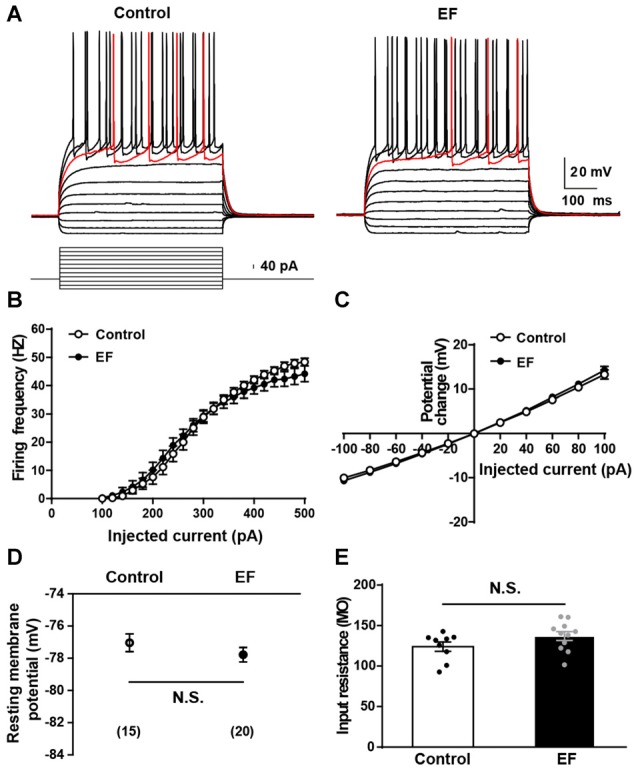
Basic electrophysiological properties of medium spiny neurons (MSNs) were normal in EF mice.** (A)** Sample traces obtained from a Control and an EF MSN in response to injection of hyperpolarizing and depolarizing current pulses. The firing rate vs. injected current **(B)** and I–V curves **(C)** were indistinguishable between control and EF mice (both Student’s *t*-test, *p* > 0.05). Resting membrane potential **(D)** and input resistance **(E)** were both comparable between two groups (both Student’s *t*-test, *p* > 0.05). All data are showed as mean ± SEM. N.S., not significant.

The alteration of basic electrophysiological properties of MSNs could affect corticostriatal synaptic plasticity. To explore the mechanisms underlying EF impairing corticostriatal synaptic plasticity, first we examined the basic electrophysiological properties of MSNs in two groups. As shown in Figure 3A, action potentials were elicited by injection of a series of current steps. Note that MSNs showed the small voltage sag in responses to hyperpolarizing currents, the inward rectification of subthreshold voltage responses, and the ramp depolarization with a delayed action potential. We found that the action potential firing frequency (Figure 3B) and I–V relationship (Figure 3C) of MSNs did not differ between two groups (both Student’s *t*-test, *p* > 0.05). In addition, the resting membrane potential (Figure 3D, Control: −77.04 ± 0.55 mV, *n* = 15 cells; EF: −77.78 ± 0.45 mV, *n* = 20 cells; Student’s *t*-test, *p* > 0.05) and input resistance (Figure 3E, Control: 124.00 ± 5.68 MΩ, *n* = 9 cells; EF: 137.10 ± 5.47 MΩ, *n* = 11 cells; Student’s *t*-test, *p* > 0.05) were similar in two groups. These results indicate that the basic electrophysiological properties of MSNs are normal in EF mice. Since EF does not affect the basic electrophysiological properties of MSNs, the impaired corticostriatal synaptic plasticity after EF is not due to the alterations of MSNs’ basic electrophysiological properties.

### sEPSC Frequency of MSNs Is Increased in Exercise-Induced Fatigue Mice

**Figure 4:**
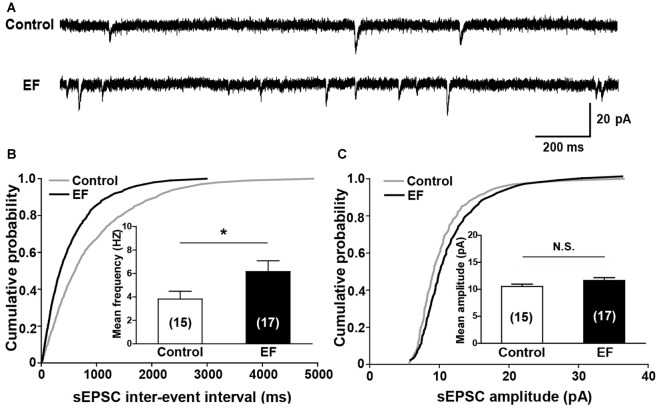
EF mice displayed normal spontaneous excitatory postsynaptic current (sEPSC) amplitude but increased sEPSC frequency.** (A)** Sample traces of sEPSCs recorded in Control and EF MSNs. **(B)** Cumulative distributions of inter-event interval and bar charts showed increased sEPSC frequency in EF mice (Kolmogorov-Smirnov test and Student’s *t*-test respectively, *p* < 0.05). **(C)** Cumulative distributions of amplitude and bar charts showed no difference in sEPSC amplitude between two groups (Kolmogorov-Smirnov test and Student’s *t*-test respectively, *p* > 0.05). All data are showed as mean ± SEM. N.S., not significant. *p < 0.05.

Next, we investigated the basal synaptic transmission in corticostriatal pathway by recording sEPSCs of MSNs (Figure 4A). The results showed that the cumulative inter-event interval distribution curve of sEPSCs in fatigue mice shifted to the left compared to control mice (Figure 4B, Kolmogorov-Smirnov test, *p* < 0.05), and the mean frequency of sEPSCs was significantly increased in EF mice (Figure 4B, Control: 3.82 ± 0.67 Hz, *n* = 15 cells; EF: 6.17 ± 0.92 Hz, *n* = 17 cells; Student’s *t*-test, *p* < 0.05), suggesting that the probability of presynaptic Glu release is enhanced after EF. Whereas no differences were observed in cumulative amplitude distribution curves (Figure 4C, Kolmogorov-Smirnov test, *p* > 0.05) and mean sEPSC amplitude between two groups (Figure 4C, Control: 10.52 ± 0.46 pA, *n* = 15 cells; EF: 11.64 ± 0.52 pA, *n* = 17 cells; Student’s *t*-test, *p* > 0.05), indicating that EF does not affect the postsynaptic AMPA receptor function in the striatum.

### PPR of MSNs Is Decreased in Exercise-Induced Fatigue Mice

**Figure 5:**
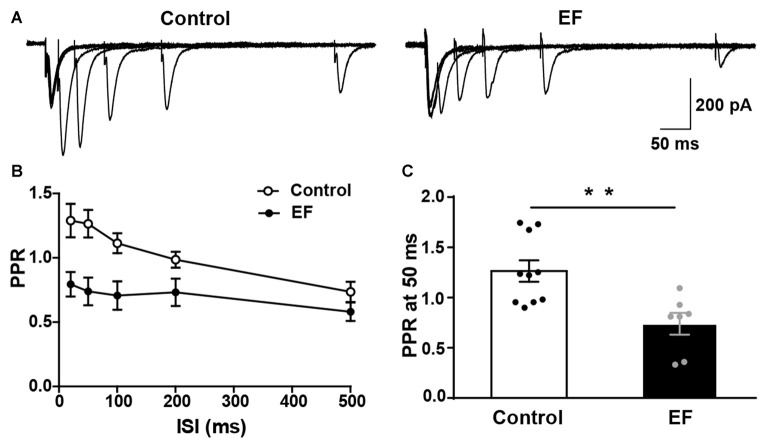
Paired-pulse ratio (PPR) of MSNs was decreased in EF mice.** (A)** Representative traces of EPSCs evoked by paired-pulse stimulation at 20, 50, 100, 200 and 500 ms inter-stimulus intervals (ISI). **(B)** Summary graph of PPR from Control and EF MSNs. **(C)** PPR at 50 ms interval was decreased in EF mice (Student’s *t*-test, *p* < 0.01). All data are showed as mean ± SEM. **p < 0.01.

In order to further verify that the striatal presynaptic Glu release was increased after fatigue, we also examined the PPR of MSNs (Figure 5A). As shown in Figures 5B,C, PPR in EF mice was significantly decreased compared to control group (PPR at 50 ms, Control: 1.27 ± 0.11, *n* = 10 cells; EF: 0.74 ± 0.11, *n* = 7 cells; Student’s *t*-test, *p* < 0.01). Since PPR is inversely proportional to the probability of presynaptic neurotransmitter release (Debanne et al., 1996), the decreased PPR in fatigue mice suggests that the presynaptic Glu release is enhanced after EF. This is consistent with the result of increased sEPSC frequency. Together, these results indicate that the striatal presynaptic Glu release is enhanced after EF.

### NMDA/AMPA Ratio of MSNs Is Decreased in Exercise-Induced Fatigue Mice

**Figure 6:**
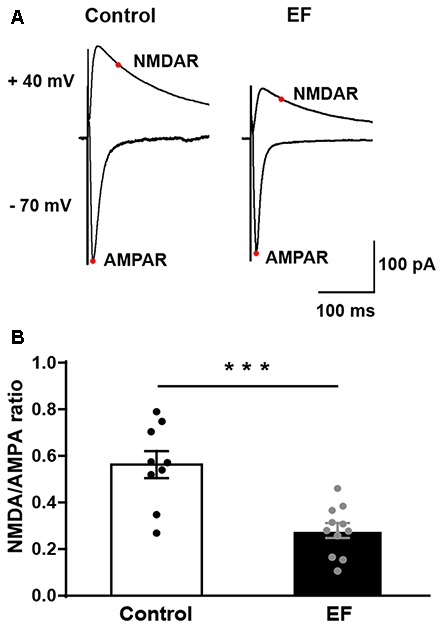
The NMDA/AMPA ratio of MSNs was decreased in EF mice. **(A)** Representative current traces recorded at + 40 mV and −70 mV from Control and EF MSNs. **(B)** NMDA/AMPA ratio was significantly decreased in EF mice (Student’s *t*-test, *p* < 0.001). All data are shown as mean ± SEM. ***p < 0.001.

To examine whether NMDA receptor function is affected by EF, we measured the ratio of NMDA receptor-mediated EPSC (current at 50 ms, V_hold_ = + 40 mV) to AMPA receptor-mediated EPSC (peak current, V_hold_ = −70 mV) in control and fatigue mice (Figure 6A). The results showed that NMDA/AMPA ratio was significantly decreased in EF mice (Figure 6B, Control: 0.56 ± 0.06, *n* = 9 cells; EF: 0.28 ± 0.03, *n* = 11 cells; Student’s *t*-test, *p* < 0.001). As mentioned above, the function of AMPA receptor was normal in EF mice, therefore, the decrease of NMDA/AMPA ratio was due to the downregulated NMDA receptor function. These results indicate that EF impairs NMDA receptor function in the striatum.

## Discussion

The goal of the present study is to examine whether EF affects the corticostriatal synaptic plasticity. Mice were forced to run until exhaustion daily for seven consecutive days on an electric treadmill to establish the EF model. EF mice exhibited deficits both in LTP and LTD, and this LTP was NMDA receptor-dependent, LTD was eCB-dependent. Investigating the mechanisms underlying impaired corticostriatal plasticity in fatigue mice, we found that EF increased sEPSC frequency, decreased PPR and reduced NMDA/AMPA ratio of MSNs, indicating that the probability of presynaptic Glu release is enhanced and NMDA receptor function is impaired after EF, while leaving basic electrophysiological properties of MSNs and sEPSC amplitude unaffected. The enhanced presynaptic Glu release and decreased NMDA receptor function may contribute to the impairment of bidirectional corticostriatal plasticity after EF.

The corticostriatal pathway is the first step of cortical information processing in the basal ganglia. Corticostriatal plasticity alters the transfer of information throughout basal ganglia circuits and may represent a key neural substrate for adaptive motor control and procedural memory (Yin and Knowlton, 2006; Kreitzer and Malenka, 2008). Unlike many other excitatory synapses that are potentiated by HFS, repeated HFS activation of cortical afferents induces LTD at corticostriatal synapses (Kheirbek, 2007; Paillé et al., 2010). HFS-induced striatal LTD is dependent upon the activation of L-type Ca^2+^ channels, group 1 metabotropic glutamate receptors (mGluRs), D2 dopamine (DA) receptors and the generation of eCBs (Choi and Lovinger, 1997; Sung et al., 2001; Kreitzer and Malenka, 2005). eCBs are released from MSNs, retrograde to the presynaptic membrane and activate the CB1 receptors on presynaptic terminals, finally lead to a decrease in the probability of presynaptic Glu release, induce the eCB-LTD. In contrast to the ease of inducing LTD at corticostriatal synapses, LTP is difficult to reliably elicit. It is NMDA receptor-dependent, and could only be induced after pharmacological manipulation, such as removal of extracellular Mg^2+^ to facilitate the opening of NMDA receptor channels (Calabresi et al., 1992; Kerr and Wickens, 2001). The molecular mechanisms underlying corticostriatal LTP remain poorly understood.

In control mice, we found that in Mg^2+^-free solution, which results in massive Ca^2+^ influx through NMDA receptor channels, HFS activation of cortical inputs induced NMDA receptor-dependent LTP in the striatum; in Mg^2+^-containing solution, HFS induced a remarkable eCB-LTD. However, in EF mice, both the LTP and LTD were significantly impaired. Further exploring the mechanisms, we found that the action potential firing frequency, I–V relationship, resting membrane potential and input resistance of MSNs were all normal in fatigue mice, indicating that EF does not affect the basic electrophysiological properties of MSNs. Besides, the sEPSC amplitude was normal, which means the postsynaptic AMPA receptor function is not changed after EF. Kamakura et al. (2005) reported that the gene expression of GluR1 was increased in the hippocampus of fatigue mice. In their study, mice were forced to swim in an adjustable-current water pool as the fatigue mouse model. Different mouse models, brain regions and test methods may be the reasons of inconsistent results between us.

Glu is the excitatory neurotransmitter in the corticostriatal pathway. Many studies have investigated whether the level of Glu is changed after EF. Because of different metabolic responses to exercise with different intensity and duration, and the different brain areas studied, the results are various. For instance, Guezennec et al. (1998) reported the Glu level was reduced after exhaustion in the striatum of rats. Świątkiewicz et al. (2017) using proton magnetic resonance spectroscopy technique showed that the Glu signal was increased in the cerebellum and hippocampus, but not changed in the striatum of rats following acute exhaustive exercise. However, Liu et al. (2011) reported that the content of Glu was significantly increased in rat’s telencephalon after EF. Similarly, Zhang et al. (2001) found that in the hypothalamus the level of Glu was increased after exhaustive exercise. In our study, whole-cell patch clamp recordings showed that the sEPSC frequency was increased and the PPR was decreased in the striatum of fatigue mice, consistently indicating that the probability of Glu release in the striatum is enhanced after EF. The enhanced Glu release may result in the deficit in eCB-LTD. In addition, we suppose that the enhanced Glu release could have a ceiling or saturating effect for LTP, decreasing the degree of potentiation of synaptic responses in EF mice.

NMDA receptor is known to be essential for corticostriatal LTP (Calabresi et al., 1992; Kerr and Wickens, 2001). By adding the NMDA receptor antagonist APV, we proved that the LTP in corticostriatal pathway was NMDA receptor-dependent. Furthermore, we found that the NMDA receptor function was downregulated in EF mice. The deficit in NMDA receptor function may lead to less Ca^2+^ influx, which is insufficient to induce LTP, consequently impair the LTP. This may be another reason for EF impairing corticostriatal LTP.

In addition to receiving glutamatergic inputs from the cerebral cortex and thalamus, striatum also receives a massive dopaminergic inputs from substantia nigra (Bolam et al., 2000; Kreitzer and Malenka, 2008). The involvement of two neurotransmitter systems is a peculiar feature of striatal plasticity, corticostriatal synaptic plasticity is regulated both by glutamatergic inputs and dopaminergic inputs. It has been shown that DA receptor activation is critical for striatal LTP and LTD induction (Centonze et al., 2001; Pisani et al., 2005). Abnormal dopaminergic inputs could affect corticostriatal synaptic plasticity and motor behavior. For example, in PD animal models, the lack of endogenous DA innervation to the striatum caused the loss of corticostriatal LTP and LTD (Calabresi et al., 2007). Further studies found that DA denervation did not alter the intrinsic membrane properties of striatal MSNs (Calabresi et al., 1993), but notably, increased the concentration and release of Glu from corticostriatal terminals (Lindefors and Ungerstedt, 1990; Calabresi et al., 1993). These observations indicate that under normal conditions, the endogenous dopaminergic inputs exert a negative modulation on glutamatergic transmission (Pisani et al., 2005). In addition, it has been reported that a reduction of DA innervation induced a loss of NMDA receptor function (Steiner et al., 2010). In our study, we also found that the intrinsic membrane properties of striatal MSNs were normal, the probability of presynaptic Glu release was increased and the NMDA receptor function was impaired after EF. Therefore, we speculate that in EF mice the DA innervation to the striatum is decreased. This is supported by numerous studies investigating the mechanisms of fatigue, which have shown that in addition to the increased 5-HT concentration, reduction in DA could contribute to EF (Foley et al., 2006; Foley and Fleshner, 2008). Increasing brain DA can delay the onset of fatigue (Bhagat and Wheeler, 1973; Cooter and Stull, 1974; Gerald, 1978), while reducing the DA activity can hasten the onset of fatigue (Derevenco et al., 1986; Davis et al., 2003). We suppose that a reduction in DA innervation to striatum could enhance the release of Glu, downregulate the NMDA receptor function, impair the corticostriatal synaptic plasticity and the activation of the basal ganglia, consequently reduce the stimulation of the motor cortex, lead to the central fatigue. Further studies about the striatal DA concentration after EF are required to confirm our hypothesis.

## Conclusion

In conclusion, we found that EF impaired the NMDA receptor-dependent LTP and eCB-LTD in the corticostriatal pathway. Further, EF enhanced the release of presynaptic Glu and downregulated postsynaptic NMDA receptor function, which may be the mechanisms underlying the impaired corticostriatal synaptic plasticity. Our results for the first time show that EF impairs the bidirectional corticostriatal synaptic plasticity, and suggest that the abnormal corticostriatal plasticity may participate in the generation and/or maintenance of fatigue.

## Author Contributions

JM conducted the study, performed the electrophysiological experiments and wrote the manuscript. HC trained the mice and contributed to electrophysiological experiments. LZ analyzed the data. JM, XL and DQ designed the study. XL and DQ edited and revised the manuscript.

## Conflict of Interest Statement

The authors declare that the research was conducted in the absence of any commercial or financial relationships that could be construed as a potential conflict of interest.

## Abbreviations

ACSF: artificial cerebrospinal fluid
AMPA: α-amino-3-hydroxy-5-methyl-4-isoxazolepropionate
CB1: cannabinoid type-1
CNS: central nervous system
DA: dopamine
EF: exercise-induced fatigue
EPSC: excitatory postsynaptic current
fEPSPs: field excitatory postsynaptic potentials
GABA: γ-aminobutyric acid
Glu: glutamate
HD: Huntington’s disease
HFS: high-frequency stimulation
ISI: inter-stimulus interval
LTD: long-term depression
LTP: long-term potentiation
mACSF: modified artificial cerebrospinal fluid
mGluR: metabotropic glutamate receptor
MSNs: medium spiny neurons
NMDA: N-methyl-D-aspartate
PD: Parkinson’s disease
PPR: paired-pulse ratio
sEPSC: spontaneous excitatory postsynaptic current.
